# Sirtuins functions in central nervous system cells under neurological disorders

**DOI:** 10.3389/fphys.2022.886087

**Published:** 2022-08-30

**Authors:** Jing Yan, Xiaole Tang, Zhi-qiang Zhou, Jie Zhang, Yilin Zhao, Shiyong Li, Ailin Luo

**Affiliations:** ^1^ Department of Anesthesiology, Tongji Hospital, Tongji Medical College, Huazhong University of Science and Technology, Wuhan, China; ^2^ Department of Anesthesiology, Sun Yat-sen University Cancer Center, Guangzhou, China

**Keywords:** sirtuins, central nervous syste, neuron, microglia, astrocyte, oligodendrocyte

## Abstract

The sirtuins (SIRTs), a class of NAD+ -dependent deacylases, contain seven SIRT family members in mammals, from SIRT1 to SIRT7. Extensive studies have revealed that SIRT proteins regulate virous cell functions. Central nervous system (CNS) decline resulted in progressive cognitive impairment, social and physical abilities dysfunction. Therefore, it is of vital importance to have a better understanding of potential target to promote homeostasis of CNS. SIRTs have merged as the underlying regulating factors of the process of neurological disorders. In this review, we profile multiple functions of SIRT proteins in different cells during brain function and under CNS injury.

## Introduction

SIRTs in mammals are a class of proteins that possess NAD + dependent deacetylase activity or ADP-ribosyltransferase activity varies from SIRT1 to SIRT7. These SIRTs are present in different tissue and subcellular localization: SIRT1 and SIRT2 are expressed in the nucleus and cytoplasm, whereas SIRT3, SIRT4, and SIRT5 are mitochondrial, and SIRT6 and SIRT7 localized only in the nucleus (Cai et al., 2016). The initial studies found Sir2 protein contributes to lifespan extension in yeasts, worms and flies in which Sir2 and its orthologs mediated caloric restriction-induced extension of lifespan in certain genetic backgrounds (Li et al., 2007). However, mice overexpressing SIRT1 did not show lifespan extension. Striking, whole-body SIRT6 transgenic mice showed lifespan extension in males (Zorrilla-Zubilete et al., 2018). Later studies indicated SIRTs’ protect brain function in virous mammalian models. Recent evidence showed that SIRTs exert anti-aging and neuroprotective effect through regulating diverse cell functions and responses to stressors (Herskovits and Guarente, 2014). Herein we review multiple functions of SIRT proteins during maintaining normal cellular function in central nervous system.

### SIRT1 in neurons in central nervous system

Sirtuin 1 (SIRT1) is a member of the nicotinamide adenine dinucleotide (NAD)+-dependent class III histone deacetylases, which is the studied one among the SIRT family (Gomes et al., 2018). Mounting Data suggest SIRT1 to be an important factor involved in a vast physiological and pathological role in brain through modulating variety of molecular signaling pathways essential for central nerves system homeostasis and normal brain function. In mammalian cells, SIRT1 located in nucleus and cytoplasm. Within the nucleus, SIRT1 regulates transcription process through promoting histone deacetylation and modulating DNA methylation. Within the cytoplasm, SIRT1 participates in regulating various cellular processes, such as cell apoptosis, oxidative stress, autophagy, endoplasmic reticulum stress and mitophagy.

Some studies suggest that cytoplasm-localized SIRT1 promotes apoptosis, while the anti-apoptotic effect attributes to the nuclear-localized SIRT1. Research indicated that SIRT1 plays a pivotal role in alleviating apoptosis. Upregulated SIRT1 alleviated manganese-induced neuronal apoptosis through activation of FOXO3a, and activated expression of SIRT1 also alleviating the apoptosis of mouse hippocampal neurons (HT22) cells through facilitating the deacetylation and phosphorylation of FOXO3a (Zhao et al., 2021a). Mostly, SIRT1 exerts neuroprotective effect through reducing apoptosis. However, nicotinamide, a pharmacological inhibitor of SIRT1, enhances neuronal survival and reduces apoptosis during acute anoxic injury, implying the benefit of SIRT1 deficiency, seemly indicate that SIRT1 might play a detrimental role (Chong and Maiese, 2008). Pharmacological or genetically downregulated SIRT1 significantly reduced ERK1/2 activation and promoted apoptotic neuron death both *in vitro* and *in vivo* of traumatic brain injury models (Zhao et al., 2012). SIRT1 deficiency in neuron leads to the acetylation of p53, a critical transcriptional factor that controls apoptotic programs and also the first nonhistone deacetylation target for SIRT1, thereby instigating neuronal death in cerebral ischemic stroke mice (Chen et al., 2014).

SIRT 1 has a vital role in autophagy stimulation through deacetylating autophagy-related proteins including FoxO, Atg5, Atg7, and Atg8 (Hariharan et al., 2010). Also, SIRT1 is a major deacetylase for BECN1 K430 and K437, which form multiple protein complexes participating in autophagy (Xu and Wan, 2022). Oxidative stress could decrease SIRT1 enzymatic activity and protein stability through promoting SIRT1 cysteinyl carbonylation on Cys482, and SIRT1-deficient cells are more sensitive to exogenous H_2_O_2_ that could induce autophagy (Zhou et al., 2022a). SIRT1 activation plays a pivotal role protecting against prion-induced neuronal death through regulating autophagy process (Jeong et al., 2013). In a cardiac arrest-related brain injury, researchers used a SIRT1 loss of function approach and found that suppression of SIRT1 compromised the neuroprotection by mild hypothermia treatment both *in vivo* and *in vitro* through mediating autophagic flux, they came up with the idea that FOXO1, a transcription factor binds to several consensus sites on the SIRT1 promoter and enables its transcription, might be the upstream of SIRT1 in this model, and further investigation is warranted (Wei et al., 2019).

Accumulating evidence has indicated that SIRT1 function is tightly related to axon genesis and neurite outgrowth. SIRT1 deficiency could cause synaptic plasticity impairment and cognitive dysfunction (Cai et al., 2016). SIRT1 regulates transcription factors, including nuclear factor-E2-related factor 2 (Nrf2), a major regulator in antioxidant defenses, to exert a neuroprotective effect by inducing neurite outgrowth in Neuro-2a cells (Duangjan et al., 2021), peroxisome proliferator-activated receptor coactivator-1α (PGC-1α), a fasting-induced transcriptional coactivator recruited during PPAR stimulation, to restore neuronal network disconnection through reversing synaptic failure in hippocampal neuron *in vitro* (Panes et al., 2020). In neonatal propofol exposure experiment, SIRT1 was tightly related to synaptic plasticity and neuronal excitability in the hippocampal CA1 region (Ma et al., 2022).

SIRT1 also take parts in physiological and pathological process correlated to neuronal cell damage such as oxidative stress, metabolic disorders and calcium homeostasis. It has been reported that SIRT1 counteracts the activation of acetyl-STAT3 (Lys685) and increase neural senescence and affects locomotor behavior (Liu et al., 2021a). SIRT1/PGC-1α pathway relieving neuronal oxidative stress through activating mitochondrial biogenesis against Aβ1-42 oligomer-induced oxidative stress (Yin et al., 2021a). As SIRT1 mediated caloric restriction, study on unhealthy-diet-induced learning and memory loss found that biogenesis related genes SIRT1 and PGC1α upregulated along with reversed high-glucose-induced oxidized cellular status, mitochondrial membrane impairment, insulin signaling inhibition and neuron damage in high-fat and high-fructose feed mice (Liu et al., 2017). Compelling investment revealed that SIRT1 provides neuroprotection by promoting calcium regulation in neurons (Stoyas et al., 2020). In a PD mice model, SIRT1 was found downregulated in neuron, which leading to FOXO1 acetylation upregulation, and subsequently increased the transcription level of type A monoamine oxidase (MAO-A), an enzyme primarily engaged in catalyzing the oxidative deamination of NA and 5-HT and leading to inactivation and degradation of both monoamines. Activation SIRT1 by resveratrol restored brain NA and 5-HT levels and attenuates the depressive-like behavior (Li et al., 2020a). The effect of SIRT1 changing the acetylation status of BMAL1 and PER2 and further affecting their activity and stability respectively, is involved in the metabolism mechanism of circadian machinery (Asher et al., 2008). Later, Aras et al. provide *in vivo* evidence that SIRT1 deficiency in neurons within the hypothalamic ventromedial nucleus are more prone to develop metabolic imbalance (Ramadori et al., 2011). What’s more, they also found that SIRT1 in neurons within the hypothalamic ventromedial nucleus convey photic inputs to entrain the biochemical and metabolic action of insulin in skeletal muscle in mice (Aras et al., 2019).

In hypoxia-ischemia rat, SIRT1 expression significantly reduced in neuron, and was related to the increased ER stress and neurotoxicity due to its action in the modulation of a wide variety of signaling pathways involved in neuroprotection (Carloni et al., 2014). In our previous studies, SIRT1 overexpression could reduce tau acetylation and thus preserve cognitive function in mice following anesthesia and surgery (Yan et al., 2020). Later, our team investigate the role of neuronal SIRT1 in developing mice exposed to volatile anesthetic sevoflurane, we found that SIRT1 epigeneticlly regulated the activity of MeCP2 and CREB, therefor affect the expression of BDNF, which is a vital factor for normal neuronal function (Tang et al., 2020).

### SIRT1 in microglia in central nervous system

Microglia are immune cells of the resident macrophages in the brain, which is continually involved in surveillance of the brain parenchyma, and providing as the first line of defense against disease-causing pathogenic stimuli. Mountings of findings indicated that SIRT1 deficiency could injury normal function of microglia (Meng et al., 2020). Mitochondria dysfunction linked neurodegeneration and neuroinflammation *via* coordinating innate and adaptive immune responses, and initiate and promoted the activation of microglia through increase reactive oxygen species (ROS). It has been reported that SIRT1 regulates PGC-1α by increasing its expression and decreasing its acetylation in microglia at least in part rescued mitochondria injury, decreased ROS production, and reduced neuroinflammatory response which ultimately ameliorated the cognitive dysfunction in chronic cerebral hypoperfusion models *in vivo* and *in vitro* (Zhao et al., 2021b). PGC-1α, a major regulator of ROS metabolism and mitochondria biogenesis, has also been proved to be downstream of SIRT1 in post-neonatal hypoxic–ischemic encephalopathy rat (Xie et al., 2021). HMGB1/NF-kB pathway also might be downstream of SIRT1 to inhibit microglia activation and proinflammatory cytokines release in subarachnoid hemorrhage rats and LPS treated BV2 cells (Wang et al., 2019; Zhang et al., 2020a; Li et al., 2020b).

Chronic and persistent activation of microglia has been proved to drive cells in the central nerves system to senescence phenotype through senescence-associated beta-galactosidase (SA β-gal) activity upregulation, growth arrest, and senescence-associated secretory phenotypes (SASPs) which involves uncontrolled secretion of proinflammatory cytokines, which exhibit detrimental effects on cells (Hardeland, 2019). SIRT1 was reduced in aging brain and associated with the impairment of learning and memory (Chaudhuri et al., 2013; Meng et al., 2020). In our previous work, we found that SIRT1 expression significantly decreased in anesthesia and surgery treated aged mice, and upregulated SIRT1 could significantly reduce microglia activation-related neuroinflammation and ameliorated cognitive impairment through regulating the expression of DNMT1 and ac-NF-κB (Yan et al., 2019). Our result is consistent with a previous study that SIRT1 deficiency in microglia contribute to aging and neurodegeneration-related cognition decline, importantly, in this article, they proved that SIRT1 exert neuroprotection through hypomethylating the specific CpG sites on IL-1β proximal promoter which is a classic inflammatory factor (Cho et al., 2015). Later, our team investigated SIRT1/NF-κB signaling in developing mice, as expected, we found that SIRT1 overexpression inhibited microglial activation and improved the long-term cognitive function through decreasing the expression of ac-NF-κB (Tang et al., 2021). In early 2005, Li Gan and his group have indicated that overexpression of SIRT1 deacetylase increased acetylation of RelA/p65 at lysine 310 and regulates the NF-κB pathway in amyloid-β peptides treated primary microglia (Meng et al., 2020). Recent research confirmed that in BV2 cells, reduced SIRT1 expression caused an increased inflammatory response in microglia termed as M1 polarization and promoted microglial migration (Yin et al., 2021b), and suppressed acetylation of NF-κB p65 subunit by SIRT1 in BV-2 microglia decreased the inflammatory factors, including TNF-α and IL-6 (Qin et al., 2020; Zhu et al., 2020). Besides, SIRT1 exerted protection against hypoxic-derived neuronal damage through regulating NF-κB (Merlo et al., 2020). And activated SIRT1/FOXO1 pathway could also reduce microglia activation and inflammatory response (Zhang et al., 2021a). SIRT1 down-regulated the level of Ac-NFκB expression and then suppressed the expression of pro-inflammatory cytokines, accompanied by the decreased activation of microglia, to withhold the cognitive function in mice (Pan et al., 2018). Furthermore, downregulated SIRT1 level prevented M2 microglial polarization and promote motor and nonmotor deficits in Parkinson’s disease (PD) mice (Yang et al., 2021).

The anti-inflammatory effect of SIRT1 has also been explored in several neurodegenerative diseases. In Parkinson’s disease models, SIRT1 signaling pathway is involved in NLRP3 inflammasome activation in microglia (Zheng et al., 2021), that has been proved again in an subarachnoid hemorrhage mice model in which SIRT1 not only involved in microglia activation, but also M2 microglial polarization (Zhang et al., 2021b). In aluminum chloride treated mice, SIRT1 regulated DDX3X-NLRP3 Inflammasome signaling pathway (Hao et al., 2021). Other substrate of SIRT1 such as NF-κB has been found changed and involved in microglial polarization in Parkinson’s disease models (Yang et al., 2021). In Alzheimer’s disease models, SIRT1 directly interacted with and deacetylated TFEB at lysine residue 116 to enhanced lysosomal function and fAβ degradation, thus attenuating amyloid plaque deposition in APP/PS1 transgenic mice (Bao et al., 2016).

SIRT1 has been found neuroprotective in other neurological disorders such as microgliopathic pain, which might be stimulated by activated microglia induced abnormal discharge of neurons, *via* its anti-inflammatory effect (Wen et al., 2021); and radiation-induced brain injury, in which SIRT1 exert protective effect through reducing oxidative stress damage, inflammation and microglial infiltration (Liu et al., 2021b); and chronic unpredictable mild stress mice model, in which SIRT1 signaling exerted antidepressant effect through modulating NLRP3 inflammasome deactivation (Tong et al., 2020); and brain injury in epilepsy and acute ethanol intoxication, in which SIRT1 prevented astrocytes and microglia activation, as well as inhibition of oxidative stress (Khan et al., 2018; Kong et al., 2020); and LPS treated neonatal mice, in which SIRT1/Nrf2 signaling pathway activation reduced LPS-induced oxidative stress damage, acute neuroinflammation, and apoptotic neurodegeneration (Shah et al., 2017). Researches also demonstrated that SIRT1 deficiency in microglia caused neuroinflammation, subsequently impaired neuronal function through apoptosis, autophagy, et al. (Liu et al., 2019).

### SIRT1 in astrocytes in central nervous system

Astrocytes perform various functions in central nerves system including immunity modulation, transmitter uptake and regulation, brain structures support, blood-brain barrier permeability regulation. Downregulation of SIRT1 is reported to be related with astrocytes activation in PD model, AD model and traumatic brain injury model (Scuderi et al., 2014; Abd El-Fatah et al., 2021; Zhang et al., 2021c; Har-Even et al., 2021; Yang et al., 2022). HIV-associated neurocognitive disorders presented in almost 50% of the infected individuals, it was founded downregulated SIRT1 expression in the HIV Tg rats with a concomitant increase in astrocyte marker (Hu et al., 2017). Astrocytic SIRT1 inhibition significantly increased the acetylation of forehead box protein O4, decreased the expression of superoxide dismutase two and catalase, and increased reactive oxygen species production *in vitro* (Cheng et al., 2014). Confocal imaging showed that SIRT1 upregulated by resveratrol increased the expression of LC3 in astrocytes around the lesion site after injury in traumatic brain injury mice (Zhang et al., 2019a). In brain injury model, elevated SIRT1 levels by resveratrol significantly increases the level of p-ERK but reduces the levels of p-JNK and p-p38 protein, thus attenuated astrocyte activation (Li et al., 2017). Also, SIRT1 upregulation was accompanied with downregulated mRNA expression of pro-inflammatory cytokines, including TNF-α and IL-1β, decreased activation of astrocytes and ameliorated blood-brain barrier disruption in ischemic brain of mice (Li et al., 2020c).

As astrocyte activation precedes extracellular Aβ deposition, an AD research focused on astrocyte function, and they found that SIRT1 could enhance the ability of astrocytes to clear Aβ, and subsequently essentially delay the formation of amyloid deposits *via* deacetylating several lysosome-related proteins and upregulate lysosome number (Li et al., 2018). In chronic cerebral hypoperfusion rat model, AMPK/SIRT1 signaling was reduced along with increased expression of STAT3/NF-κB pathway (Li et al., 2020d). Other substrate of SIRT1 in astrocytes has been revealed. SIRT1 plays a protective role against astrocyte activation through interaction with Dnajb1 and modulate the deacetylation and ubiquitination of Dnajb1 *in vivo* and *in vitro* model of traumatic brain injury (Zhang et al., 2022). In cerebral ischemia/reperfusion injury model, SIRT1 directly mediated the PGC-1α/PPARγ pathway in astrocytes, attenuated oxidative stress injury and therefore reversed the neurological deficit (Zhou et al., 2022b).

### SIRT1 in oligodendrocytes in central nervous system

Oligodendrocyte precursors differentiate into mature myelin forming oligodendrocytes to preserve the balance between demyelination and remyelination, which keep the myelin sheath renewal and the axons in a normal status, thus support neuron nutrition in the central nervous system (Prozorovski et al., 2019; Hisahara et al., 2021). As mature and terminally differentiated cells that form myelin sheaths around axons, oligodendrocyte are found predominantly, but not exclusively, in CNS white matter, and could produce myelin sheaths that allow “saltatory” action potential propagation, therefor greatly increases conduction velocity, speeding the efficiency of communication between CNS neurons (Zhou et al., 2021). In an exercise training and dietary fat interplay mice model, SIRT1, PGC-1α and antioxidant enzymes were increased and contribute to mitochondrial activity in oligodendroglia in response to higher levels of reactive oxygen species (Yoon et al., 2016). SIRT1 regulated oligodendrocyte regeneration *via* deacetylating retinoblastoma in the Rb/E2F1 complex, leading to E2F1 dissociation in neonatal brain injury mice (Jablonska et al., 2016). In a mouse model of multiple sclerosis, increased oligodendrocyte generation and decreased apoptosis were consistent with increased SIRT1 expression (Mojaverrostami et al., 2020). It was observed that SIRT1 co-localizes with surviving oligodendrocytes in multiple sclerosis plaques, then the research group found a significant reduction in phospho-SIRT1 and SIRT1 expression during oligodendrocytes differentiation, associated with decreased expression of H3K9me3 and increased cyclin D1 (Tatomir et al., 2020). Taken together, SIRT1 promoted oligodendrocytes differentiation and regeneration in the CNS.

## SIRT2 in central nervous system cells

SIRT2 is predominantly cytosolic and shuttle between the cytoplasm and nucleus. Several studies indicated an important role of SIRT2 in cognitive and brain function. SIRT2 localized to the outer and juxtanodal loops in the myelin sheath and has a counterbalancing role on oligodendroglial differentiation (Li et al., 2007). Fang N found that SIRT2 translocated into the nuclei, epigeneticly downregulated PDGFRα expression and facilitate the differentiation of oligodendroglial cell line (Fang et al., 2019).

ATP homeostasis in axons is highly vulnerable to bioenergetic failure, and is required in neuron function. *In vivo* research revealed that of SIRT2 transcellular oligodendrocyte-to-axon delivery enhances ATP production by deacetylating mitochondrial proteins and enhanced axonal energy in mice (Chamberlain et al., 2021). SIRT2 is highly expressed in oligodendrocytes and be released within exosomes. Still, there are few researches found SIRT2 expressed in neurons. Genetic or pharmacologic SIRT2 inhibition reduced sterol levels *via* decreased nuclear SREBP-2 trafficking and showed neuroprotective effect in a striatal neuron model of HD (Luthi-Carter et al., 2010).

On the other hand, SIRT2 inhibition has been proved beneficial in some neurodegenerative mouse model. Pais TF reported, for the first time, that SIRT2 is also expressed in microglia and the absence of SIRT2 enhanced microglia activation associated with a proinflammatory phenotype induced by LPS in BV2 cells (Pais et al., 2013). Compelling evidence indicated that SIRT2 accumulates in several microglia models. SIRT2 increased lipopolysaccharide-induced microglial activation *in vivo* and *in vitro* through regulation of MAKP signaling and inflammatory response including a major inflammation transcription regulator, the p65 subunit of NF-kB (Chen et al., 2015a; Jiao et al., 2020),besides, SIRT2 showed potential effect on intracellular ATP levels through poly (ADP-ribose) polymerase (PARP) activation in microglia cell line (Li et al., 2013). Animal study showed that SIRT2 also promote sevoflurane-induced learning and memory deficits in the developing rat brain. SIRT2 inhibitor, AK7, pretreatment protected against sevoflurane-mediated cognitive impairments possibly by enhancing the proportion of M2-phenotype microglia (Wu et al., 2020). However, AK7, the brain-permeable SIRT2 inhibitor, which showed neuroprotective effect in neurodegenerative disease including Parkinson’s disease and Huntington’s disease by protecting dopaminergic neurons against aSyn-induced neurotoxicity *in vitro* and promotes long-term survival of dopaminergic neurons *in vivo*, does not show beneficial effects in amyotrophic lateral sclerosis and cerebral ischemia mouse model (Chen et al., 2015b). Furthermore, studies have reported that AK7 is not beneficial under conditions where the control of the microglial response is crucial for neuronal survival, and the anti-inflammatory properties of SIRT2 through post-translational deacetylation of p65 might be a underlying mechanism (Romeo-Guitart et al., 2018). Not only in microglia, in rat primary astrocytes, SIRT2 inhibition partly induced cellular senescence through increase senescence-associated β-galactosidase (SA-β-gal) activity, senescence-associated secretory phenotypes and cell cycle-related proteins (Bang et al., 2019).

### SIRT3 in neurons in central nervous system

Sirtuin-3 (SIRT3), localized in the mitochondria, is a NAD^+^-dependent deacetylase. SIRT3 exerts protection against the progression of cognitive dysfunction through regulates mitochondrial energy metabolism, antioxidant mechanisms, inflammation, autophagy, and cell death processes *via* targeting of the involved enzymes (Almalki et al., 2021). Mitochondria are small double-membrane organelles which could provide energy for cellular functions. Mitochondrial dysfunction has reported as a significant determinant in brain dysfunction pathophysiological processes (Song et al., 2013). SIRT3 can deacetylate and activate LKB1 (liver kinase B1), and active LKB1 can phosphorylate AMPK to activate AMPK (Liu et al., 2020). In ischemic-injured PC12 cells, promoting SIRT3/AMPK pathway significantly increased cell survival, decreased apoptosis rate and increased the mitochondrial autophagy (Li et al., 2021). It has been reported that increased SIRT3 providing neuroprotective effects *via* enhancing mitochondrial function, increase neuron survival and decrease the number of apoptotic neurons in cerebral ischemia rat through activation of the SIRT3/AMPK/mTOR pathway (Liu et al., 2020). In addition, SIRT3 alleviated cell death and promoted cell viability of the HT22 cells through preservation of mitochondrial function (Lin et al., 2021). Cerebral ischemia-reperfusion injury caused neuron apoptosis, SIRT3 overexpression attenuated neuron apoptosis possibly through blocking caspase-9-dependent mitochondrial apoptotic signals and suppressed mitochondrial fission *via* activating the Wnt/β-catenin pathway *in vitro* and *in vivo* (Zhao et al., 2018).

SIRT3 played a positive role in promoting autophagy in HT22 cells to resist from the neurotoxicity induced by Aβ1–42 oligomers through increasing Beclin-1 and LC3-II expression, as well as led to the p62 expression (Zhang et al., 2020b). In a subarachnoid hemorrhage mice model, SIRT3 exerted anti-apoptotic effect on neuron through deacetylated SOD to decrease neuron lose (Zhang et al., 2019b). In Aβ42 treated APPTG mice, SIRT3 activity and expression was found to be decreased and be associated with hippocampal neuron cell apoptosis and cognition deficit (Liu et al., 2021c). Besides, SIRT3 has been reported as neuroprotective in ALS models (Harlan et al., 2020). However, the underline mechanism of SIRT3 in these processes still needs further investment. In a high-fat diet mice model, the hippocampus plasticity was reduced and spatial learning function was compromised, SIRT3 upregulation could decrease hippocampal neuron oxidative stress and apoptosis to attenuates high-fat diet-associated cognitive dysfunction through acetylate antioxidative MnSOD (Shi et al., 2018). In an ischemia research, SIRT3 SUMOylation triggered by SENP1 subsequently results in increased levels of COX1, SOD2, and IDH2 protein acetylation, which caused oxidative stress and mitochondrial dysfunction *in vitro* and *in vivo* (Cai et al., 2021).

In a study of SIRT3-depleted mice, the behavioral phenotypes have been explored, they found that locomotion, anxiety, and recent memory of the mice remain normal, but the remote memory was impaired, this consist with impaired long-term potentiation in the anterior cingulate cortex which might due to neuronal loss (Kim et al., 2019). It has been reported that SIRT3 reduction caused aggravated GABAergic neuron loss and neuronal network hyperexcitability in Sirt3^+/−^AppPs1 mice (Cheng et al., 2020). Besides, SIRT3 was found increased in substantia nigra after physical exercise which might be related to an increase in expression of the protective Ang 1-7/Mas axis and inhibition of the Ang II/AT1 axis (Munoz et al., 2018). Although these results suggest that SIRT3 is protective in neuronal function in various manners including neuronal apoptosis, autophagy, mitochondrial function preservation, neuronal network hyperexcitability, future investigations are necessary to confirm the underline signaling pathways.

Research on cardiomyopathy revealed that PGC-1α plays a role in moderating SIRT3, which in turn deacetylates enzymes to control antioxidants and the metabolization of mitochondrial energy (Zhu et al., 2020). Similarly, PGC-1α/SIRT3 pathway have been reported neuroprotective in hippocampus by preventing damage to mitochondria and preventing cell apoptosis and neuron loss (Cheng et al., 2021). As we have discussed in 1.1, SIRT1 has been proved to be downstream of SIRT1, thus SIRT1/PGC-1alpha/SIRT3 pathway might play an essential role in mitochondrial dysfunction, oxidative stress and related neurological deficits. (Cheng et al., 2021).

### SIRT3 in microlgia in central nervous system

SIRT3 overexpression in mouse primary microglia prevented the microglia senescence through decreasing the expression of mitochondrial antioxidant enzymes (Thangaraj et al., 2021). In a study of PD cell model, they found that decreased expression of SIRT3 underlines the pathological process of microglia activation resulted oxidative stress injury, membrane potential depolarization and mPTP opening mediated cell apoptosis in dopaminergic neurons (Jiang et al., 2019). In addition, SIRT3 exerted an anti-apoptotic effect in LPS-treated BV2 cells through suppressing the transcription of SRV2 *via* the Mst1-JNK pathway and thus suppressed mitochondrial fission. (Zhou and Jiang, 2019) Besides, Yun Yuan and his group found that SIRT3 expression could be inhibited by Notch signaling pathway interference in microglia, and that SIRT1 and Notch synergistically regulate microglia activation including TNF-α production (Guo et al., 2021). Several SIRT3 substrates have been reported involved in mitochondrial biogenesis and dynamism pathways dependent pathological mechanisms (Liu et al., 2021d). In mouse primary microglial, overexpression of SIRT3 prevented HIV Trans-activator of transcription mediated imbalance of mitochondrial oxidative stress and induction of senescence phenotype (Thangaraj et al., 2021).

In an ischemia animal model, SIRT3 expression was found to be increased in macrophages, and increased SIRT3 promoted microglial N9 cells migration by upregulating CX3CR1 (Cao et al., 2019). Activated microglia caused uncontrolled production of ROS which forms an important basis for microglia-mediated neurodegeneration and associated cognition deficit. It was reported that microglial SIRT3 upregulation facilitates the increase in Foxo3a expression and its nuclear localization to carry out its antioxidant-mediated attenuation of ROS. The researchers reassured SIRT3 as the upstream regulator of Foxo3a in microglia using Sirt3^−/−^ mouse (Rangarajan et al., 2015). In 2021, Yu-Qing Wu and his group found that SIRT3 suppressed hippocampal neuroinflammation and exert protection in anesthesia/surgery-induced cognitive decline in aged mice through ameliorating the anesthesia/surgery-induced mitochondrial oxidative stress response, microglia activation and neuroinflammation (Liu et al., 2021d). In a study of Alzheimer’s Disease, researchers found that SIRT3 deficiency caused microglia activation following exposure to a combination of high glucose and palmitic acid using SIRT3 shRNA Lentiviral particles in BV2 cells, and enhanced microglial and endothelial interactions which may leading to BBB breakdown, and thus caused cognitive dysfunction, however, the mechanism of SIRT3 deficiency under microglial and endothelial interactions has not been further exacerbated (Tyagi et al., 2021).

### SIRT3 in astrocytes and oligodendrocytes in central nervous system

It was reported that SIRT3 mediated the inhibitory effect of adjudin in astrocyte activation and glial scar formation in mouse model of transient middle cerebral artery occlusion and in primary astrocytes (Yang et al., 2017). Knockdown of SIRT3 using specific siRNA (Si-Sirt3) partially reserved the effects of AMPAR antagonist on neuronal injury and BBB function *in vitro* neurovascular unit (NVU) system including neurons, astrocytes, and brain microvascular endothelial cells (Chen et al., 2021). what’s more, SIRT3 also act as a factor regulating non-cell-autonomous neuronal death through glia. SIRT3 overexpression suppressed oxidative stress-induced neuronal toxicity in SH-SY5Y cells while SIRT3 deficiency promoted rotenone- or H_2_O_2_-induced neuronal toxicity in differentiated SH-SY5Y cells (Lee et al., 2021).

Seldom studies focusing on investigating the role of SIRT3 in astrocytes and oligodendrocytes. Further study is required to determine roles of SIRT3 in the two types of glia cells.

## SIRT4/5 in central nervous system cells

SIRT4 and SIRT5 both localize in mitochondria. SIRT4 is an ADP-ribosyltransferase whereas SIRT5 is a desuccinylase and demalonylase with weak deacetylase activity (Kida and Goligorsky, 2016). In hepatic encephalopathy, overexpression SIRT4 in astrocyte inhibited GDH2 activity and restored mitochondrial respiration (Drews et al., 2020), which suggests SIRT4 exert a protective effect in astrocyte. In an AD model, the expression of SIRT5 was significantly decreased, along with suppressed autophagy in neuron. Overexpression of SIRT5 in glia cells repressed microglia and astrocyte activation, and restored oxidative stress-induced brain damage (Wu et al., 2021). Currently investigations focused on SIRT4 and SIRT5 in CNS are rare.

## SIRT6 in central nervous system cells

SIRT6 is a chromatin-associated protein that stabilizes genomes and prevents cell premature senescence. In mice cortical and hippocampal primary neurons, SIRT6 is closely related with synaptic function, neuronal maturation and neuronal survival (Cardinale et al., 2015). Another study indicated that SIRT6 levels affect synaptic plasticity and neuronal survival *via* regulating Akt-GSK3β signaling, moreover, upregulated SIRT6 is related to depression-like behavior, which indicated that SIRT6 might exert neurotoxic effect on neuron (Mao et al., 2017) Another study on PD suggest that SIRT6 plays a pathogenic and pro-inflammatory role (Nicholatos et al., 2018). On the other hand, decreased SIRT6 expression has been found in the spinal cord of amyotrophic lateral sclerosis (ALS) patients, enhancing the activity or expression of SIRT6 abrogates its neurotoxicity in cell culture models of ALS (Harlan et al., 2020). In an ischemic stroke model, LPS-stimulated inflammatory response in primary mouse microglia can be inhibited by SIRT6 activation (He et al., 2021). The dual effect of SIRT6 in central nervous system may due to different pathological context. Currently it is not fully elucidated whether SIRT6 is protective in CNS.

## SIRT7 in central nervous system cells

SIRT7 is the only SIRT protein localized predominantly in the nucleoli (Wang et al., 2020). In cerebral ischemia/reperfusion injury cell model, overexpression of SIRT7 protect neurons possibly through regulating p53-mediated proapoptotic signaling pathway (Lv et al., 2017). SIRT7 knockdown mice proved slight protective effect through regulating cell differentiation and cytokine production *via* reducing peripheral IFN-γ production and failed accumulation of regulatory T cells in the CNS. Besides, SIRT7 promoted adult-born neurons survival but had no effect on hippocampal neurons proliferation (Burg et al., 2018). Future investigations are need to clarify the SIRT7 function in CNS.

## Discussion

The fact that sirtuins play important roles in maintaining normal cellular function to keep the homeostasis of CNS, makes them an attractive candidate to be fully investigated in central nervous system cells. As described above, the sirtuins are involved in regulating essential cell function including oxidative stress, cell mitigation, apoptosis, mitochondrial function, et al. The majority of the mammalian sirtuins exert neuroprotective effect in the cells. Some of their regulation have dual effect on cell fate under different pathological settings. Still, sirtuins mechanism has not been fully revealed, further studies are required to uncover the mysteries of sirtuins in the brain. Taken together, the apparent protective effects of sirtuins in neuron, astrocyte, microglia and Oligodendrocyte support that seven SIRT proteins synergistically regulated cell function *via* distinct mechanisms to enhance central nervous systerm homeostasis.
